# Supplementary figures and images for: Single-step in vitro reconstitution of the Escherichia coli ribosome mediated by two GTPase factors, EngA and ObgE
Source: eLife. 2026 Jun 18;15:RP109916. doi: 10.7554/eLife.109916 (PMC13278734; doi:10.7554/eLife.109916)

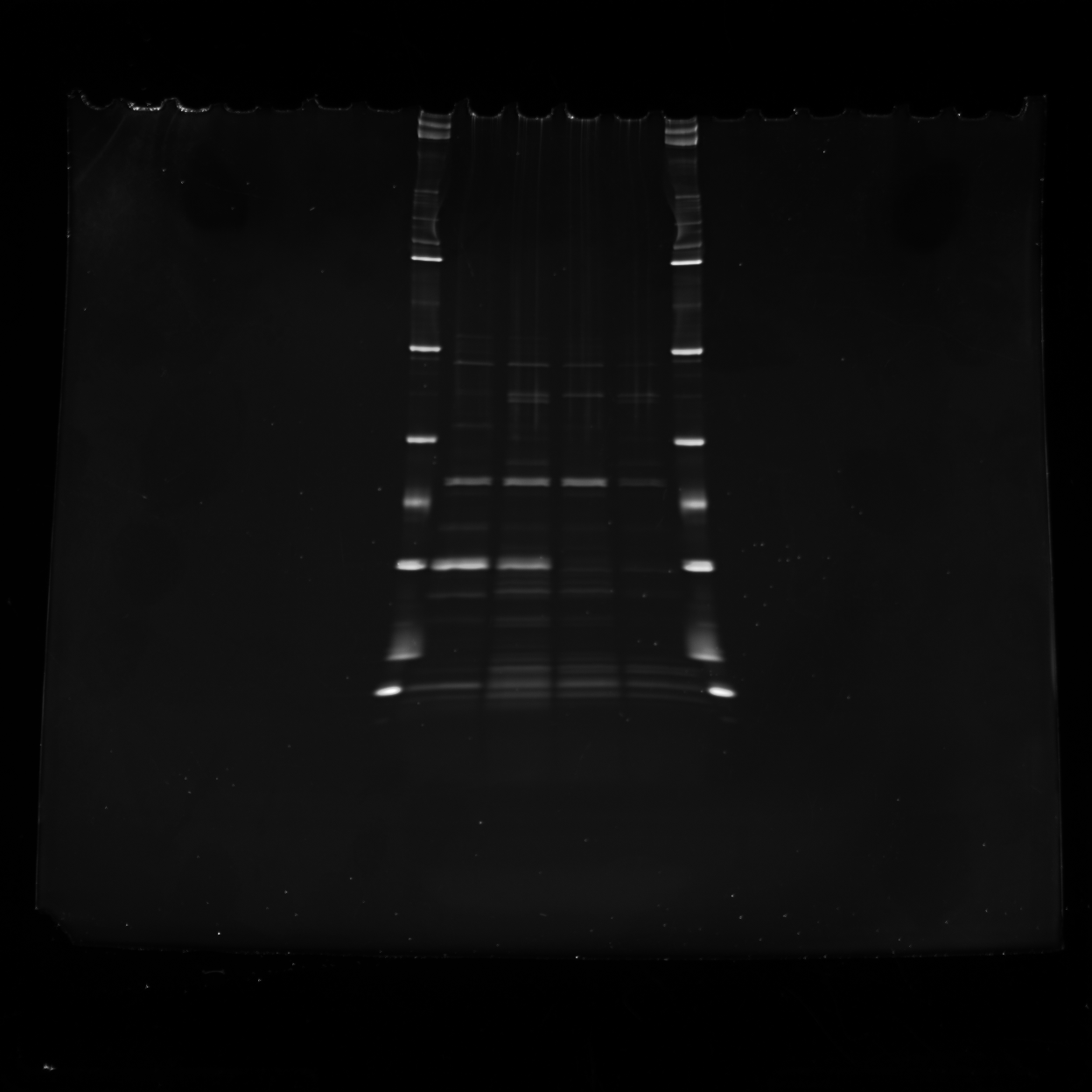

Supplement: Figure 7—source data 1. [file elife-109916-fig7-data1.zip › Figure7-SourceData1.tif]

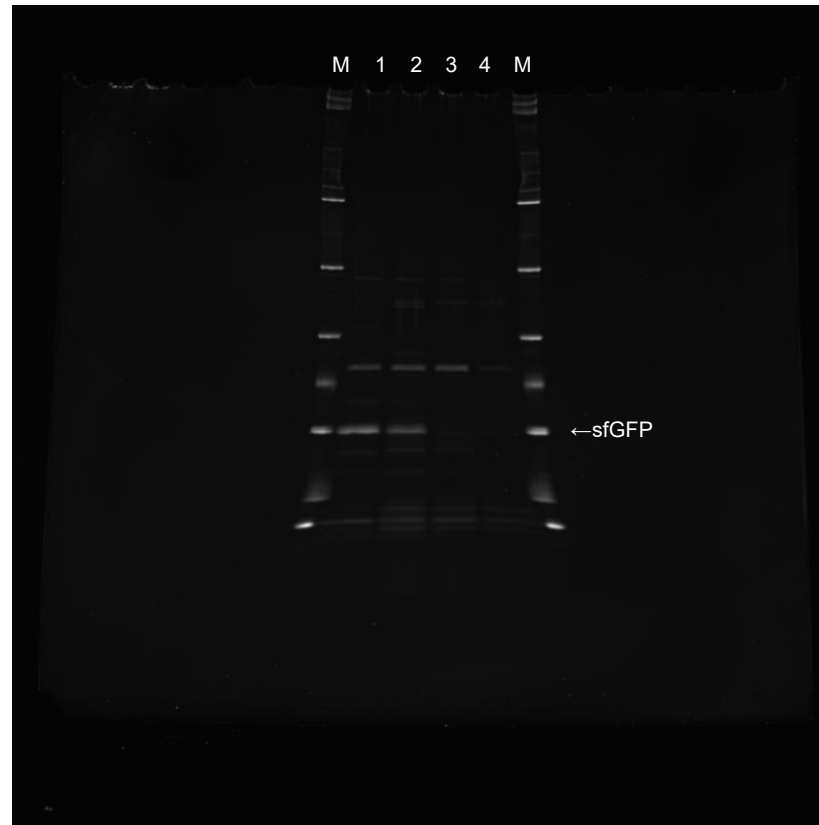

Supplement: Figure 7—source data 2. [file elife-109916-fig7-data2.zip › Figure7-SourceData2.pdf]
