# Supplementary material for: Single-step in vitro reconstitution of the Escherichia coli ribosome mediated by two GTPase factors, EngA and ObgE
Source: eLife. 2026 Jun 18;15:RP109916. doi: 10.7554/eLife.109916 (PMC13278734; doi:10.7554/eLife.109916)
Supplement: Source data 3. [file elife-109916-data3.docx]

**Source Data 3. DNA sequences of the plasmids used in this study.**

Underlined: T7 promoter or T7 terminator sequence.

Blue: leader sequence containing His-tag.

Green: SUMO protein.

Red: ribosome biogenesis factors.

Purple: ORF of tagged sfGFP and DHFR

>BipA with His-tag in pET15b

TAATACGACTCACTATAGGGGAATTGTGAGCGGATAACAATTCCCCTCTAGAAATAATTTTGTTTAACTTTAAGAAGGAGATATACCATGGGCAGCAGCCATCATCATCATCATCACAGCAGCGGCCTGGTGCCGCGCGGCAGCCATATGATCGAAAAATTGCGTAATATCGCCATCATCGCGCACGTAGACCATGGTAAAACCACCCTGGTAGACAAGCTGCTCCAACAATCCGGTACGTTCGACTCTCGTGCCGAAACCCAAGAGCGCGTGATGGACTCCAACGATTTGGAGAAAGAGCGTGGGATTACCATCCTCGCGAAAAACACCGCTATCAAATGGAATGATTACCGTATCAACATCGTTGATACCCCGGGGCACGCCGACTTCGGTGGTGAAGTTGAACGTGTAATGTCCATGGTAGACTCAGTGCTGCTGGTGGTTGACGCATTTGACGGCCCGATGCCGCAAACGCGCTTCGTAACCAAAAAAGCGTTTGCTTACGGCCTGAAGCCGATTGTTGTTATCAACAAAGTTGACCGCCCTGGCGCGCGTCCTGATTGGGTTGTGGATCAGGTATTCGATCTGTTCGTTAACCTCGACGCGACCGACGAGCAGCTGGACTTCCCGATCGTTTACGCTTCTGCGCTGAACGGTATCGCGGGTCTGGACCACGAAGATATGGCGGAAGACATGACCCCGCTGTACCAGGCGATTGTTGACCACGTTCCTGCGCCGGACGTTGACCTTGACGGTCCGTTCCAGATGCAGATTTCTCAGCTCGATTACAACAGCTATGTTGGCGTTATCGGCATTGGCCGCATCAAGCGCGGTAAAGTGAAGCCGAACCAGCAGGTCACTATCATCGATAGCGAAGGCAAAACCCGCAACGCGAAAGTCGGTAAAGTGCTGGGCCACCTCGGTCTGGAACGTATCGAAACCGATCTGGCGGAAGCTGGCGATATCGTTGCGATCACGGGCCTTGGCGAACTGAACATTTCTGACACCGTTTGCGACACGCAAAACGTTGAAGCGCTGCCGGCACTCTCCGTTGATGAGCCGACCGTTTCTATGTTCTTCTGCGTTAACACCTCGCCGTTCTGCGGTAAAGAAGGTAAGTTCGTAACGTCTCGTCAGATCCTGGATCGTCTGAACAAAGAACTGGTACACAACGTTGCGCTGCGCGTAGAAGAAACCGAAGACGCCGATGCGTTCCGCGTTTCTGGTCGTGGCGAACTGCACCTGTCTGTTCTGATCGAAAACATGCGTCGTGAAGGTTTCGAACTGGCGGTATCCCGTCCGAAAGTTATCTTCCGTGAAATCGACGGTCGTAAACAAGAGCCGTATGAAAACGTGACTCTGGACGTTGAAGAACAGCATCAGGGTTCTGTAATGCAGGCGCTGGGCGAACGTAAAGGCGACCTGAAAAACATGAATCCAGACGGTAAAGGCCGCGTACGTCTCGACTACGTGATCCCAAGCCGTGGTCTGATTGGCTTCCGTTCTGAGTTCATGACCATGACTTCCGGTACTGGTCTGCTGTACTCCACCTTCAGCCACTACGACGACGTACGTCCGGGTGAAGTGGGTCAGCGTCAGAACGGCGTACTGATCTCTAACGGTCAGGGTAAAGCGGTCGCGTTCGCGCTGTTCGGTCTGCAGGATCGCGGTAAGCTGTTCCTCGGTCACGGTGCAGAAGTTTACGAAGGTCAGATTATCGGTATTCATAGCCGCTCTAACGACCTGACTGTAAACTGCCTGACCGGTAAGAAACTGACCAACATGCGTGCTTCCGGTACTGACGAAGCCGTTGTTCTGGTTCCGCCTATCCGCATGACTCTGGAACAAGCTCTGGAGTTCATCGATGATGACGAACTGGTAGAAGTGACTCCGACCTCTATCCGTATTCGTAAACGTCACCTGACGGAAAACGATCGTCGCCGCGCCAACCGCGCACCGAAAGACGATTAAGGATCCGGCTGCTAACAAAGCCCGAAAGGAAGCTGAGTTGGCTGCTGCCACCGCTGAGCAATAACTAGCATAACCCCTTGGGGCCTCTAAACGGGTCTTGAGGGGTTTTTTG

>EngB with His-tag in pET15b

TAATACGACTCACTATAGGGGAATTGTGAGCGGATAACAATTCCCCTCTAGAAATAATTTTGTTTAACTTTAAGAAGGAGATATACCATGGGCAGCAGCCATCATCATCATCATCACAGCAGCGGCCTGGTGCCGCGCGGCAGCCATATGACTAATTTGAATTATCAACAGACGCATTTTGTGATGAGTGCGCCTGATATTCGCCACCTACCTTCCGATACCGGAATTGAAGTGGCTTTTGCAGGCCGTTCCAACGCAGGTAAATCCAGCGCGCTGAACACGCTGACTAACCAGAAAAGCCTGGCTCGTACCTCAAAAACCCCAGGGCGCACCCAGCTTATCAACCTGTTTGAAGTGGCTGACGGCAAGCGTCTGGTTGACTTGCCTGGGTACGGTTATGCGGAAGTCCCGGAAGAGATGAAGCGCAAATGGCAGCGTGCGCTCGGCGAATACCTCGAAAAACGTCAGAGCCTGCAAGGTCTGGTGGTGCTAATGGATATTCGCCATCCGCTGAAAGATTTGGATCAGCAGATGATTGAGTGGGCGGTAGACAGCAATATCGCCGTTCTGGTGCTGCTGACCAAAGCGGACAAACTGGCAAGCGGCGCACGTAAAGCGCAATTGAATATGGTGCGTGAAGCTGTACTGGCGTTTAACGGTGATGTGCAGGTTGAAACGTTTTCTTCGTTGAAGAAACAAGGCGTGGACAAGCTGCGGCAGAAACTGGATACCTGGTTTAGCGAGATGCAGCCTGTAGAAGAAACGCAGGACGGCGAATAAGGATCCGGCTGCTAACAAAGCCCGAAAGGAAGCTGAGTTGGCTGCTGCCACCGCTGAGCAATAACTAGCATAACCCCTTGGGGCCTCTAAACGGGTCTTGAGGGGTTTTTTG

>HflX with His-tag in pET15b

TAATACGACTCACTATAGGGGAATTGTGAGCGGATAACAATTCCCCTCTAGAAATAATTTTGTTTAACTTTAAGAAGGAGATATACCATGGGCAGCAGCCATCATCATCATCATCACAGCAGCGGCCTGGTGCCGCGCGGCAGCCATATGTTTGACCGTTATGATGCTGGTGAGCAGGCGGTACTGGTACACATCTATTTTACGCAAGACAAAGATATGGAAGACCTCCAGGAGTTTGAATCTCTGGTCTCTTCCGCCGGTGTCGAAGCATTGCAGGTGATTACCGGTAGCCGTAAAGCGCCGCACCCAAAGTATTTTGTAGGTGAAGGTAAAGCAGTTGAAATTGCGGAAGCTGTCAAAGCGACGGGTGCTTCGGTCGTTCTTTTTGACCATGCCCTGAGCCCGGCGCAAGAGCGTAACCTGGAGCGTTTGTGCGAGTGTCGTGTTATCGACCGCACCGGCCTTATTTTAGATATTTTCGCCCAACGTGCGCGTACCCATGAGGGTAAGTTGCAGGTTGAGCTGGCGCAGCTGCGCCATCTGGCTACGCGCCTGGTGCGTGGCTGGACCCACCTTGAAAGACAGAAAGGCGGGATAGGTTTGCGTGGTCCGGGTGAAACCCAGCTCGAAACCGACCGTCGTTTGTTGCGTAATCGCATCGTGCAGATACAGTCGCGCCTGGAAAGAGTTGAAAAGCAGCGTGAGCAGGGGCGGCAATCGCGTATCAAAGCCGACGTTCCTACTGTTTCGCTGGTGGGATATACCAACGCCGGTAAATCTACCCTTTTCAATCGCATCACCGAAGCGCGGGTCTACGCGGCAGACCAGTTGTTTGCCACCCTCGACCCGACGTTGCGGCGTATTGACGTTGCAGATGTCGGTGAAACCGTACTTGCAGATACCGTAGGGTTTATTCGCCACCTGCCGCACGATCTGGTGGCGGCATTTAAAGCCACGTTACAAGAGACGCGGCAAGCCACATTACTGCTGCACGTCATTGATGCGGCGGATGTGCGTGTACAAGAAAACATCGAAGCGGTGAATACGGTTCTTGAAGAGATCGACGCTCACGAGATCCCAACCCTGCTGGTGATGAACAAGATCGATATGCTGGAAGATTTCGAACCGCGTATTGATCGGGACGAAGAGAACAAACCGAACCGTGTCTGGCTTTCCGCACAGACCGGAGCGGGGATACCACAGCTTTTTCAGGCTTTGACGGAGCGGCTTTCCGGCGAGGTGGCGCAGCATACATTGCGTCTGCCACCGCAGGAAGGGCGTCTGAGAAGTCGTTTTTATCAGCTTCAGGCAATAGAAAAAGAGTGGATGGAGGAGGACGGCAGCGTAAGTCTGCAAGTTCGTATGCCGATCGTTGACTGGCGTCGCCTCTGTAAACAAGAACCGGCGTTGATCGATTACCTGATCTAAGGATCCGGCTGCTAACAAAGCCCGAAAGGAAGCTGAGTTGGCTGCTGCCACCGCTGAGCAATAACTAGCATAACCCCTTGGGGCCTCTAAACGGGTCTTGAGGGGTTTTTTG

>LepA with His-tag in pET15b

TAATACGACTCACTATAGGGGAATTGTGAGCGGATAACAATTCCCCTCTAGAAATAATTTTGTTTAACTTTAAGAAGGAGATATACCATGGGCAGCAGCCATCATCATCATCATCACAGCAGCGGCCTGGTGCCGCGCGGCAGCCATATGAAGAATATACGTAACTTTTCGATCATAGCTCACATTGACCACGGTAAATCGACGCTGTCTGACCGTATTATCCAGATCTGCGGTGGCCTGTCTGACCGTGAAATGGAGGCGCAGGTTCTCGATTCCATGGATCTTGAGCGTGAGCGTGGCATTACCATCAAAGCGCAAAGCGTGACGCTGGACTACAAAGCGTCTGACGGCGAAACCTATCAGCTTAACTTTATCGACACCCCGGGCCACGTAGACTTCTCCTATGAAGTTTCCCGTTCGCTGGCTGCCTGTGAAGGTGCATTGCTGGTGGTCGACGCCGGGCAGGGCGTAGAAGCGCAAACCCTGGCAAACTGCTACACCGCCATGGAAATGGATCTCGAAGTTGTGCCGGTACTGAACAAGATTGACCTGCCGGCAGCCGATCCTGAACGCGTGGCGGAAGAAATTGAAGATATCGTCGGCATCGACGCCACCGACGCGGTGCGCTGTTCAGCGAAAACCGGCGTTGGTGTGCAGGACGTTCTCGAACGTCTGGTGCGCGACATTCCGCCGCCGGAAGGCGATCCGGAAGGCCCGTTGCAGGCACTAATTATCGACTCATGGTTCGACAACTACCTGGGCGTTGTTTCACTTATCCGTATTAAAAACGGCACCCTGCGTAAGGGCGACAAAGTGAAAGTCATGAGTACCGGGCAGACCTATAACGCCGACCGTCTGGGCATCTTCACGCCGAAACAGGTTGACCGCACTGAACTGAAATGTGGCGAAGTAGGCTGGCTCGTATGTGCGATTAAAGATATCCACGGCGCTCCAGTCGGCGATACCTTAACGCTGGCGCGTAATCCGGCAGAAAAGGCGCTGCCTGGCTTTAAGAAAGTCAAACCGCAGGTATACGCCGGTCTGTTCCCGGTAAGTTCCGACGACTATGAAGCCTTCCGTGACGCGCTGGGTAAACTCAGCCTGAACGATGCCTCACTGTTCTATGAGCCGGAAAGCTCCAGCGCGCTGGGCTTTGGTTTCCGCTGCGGCTTCCTCGGCCTGCTGCACATGGAGATCATCCAGGAACGTCTGGAACGTGAATACGATCTGGATCTGATCACCACTGCGCCGACCGTAGTGTATGAAGTTGAAACCACGTCAAGAGAAGTTATCTACGTCGACAGCCCATCCAAGCTGCCTGCGGTAAATAACATCTACGAACTGCGCGAGCCGATTGCAGAGTGTCACATGCTGCTGCCGCAGGCATATCTCGGCAACGTTATTACGTTGTGCGTAGAAAAACGCGGCGTGCAGACCAATATGGTTTACCACGGTAATCAGGTGGCGCTGACGTACGAGATCCCGATGGCGGAAGTGGTGCTCGACTTCTTCGATCGCCTGAAATCTACCTCGCGTGGTTATGCGTCTCTGGATTACAACTTCAAGCGCTTCCAGGCGTCCGACATGGTACGTGTAGACGTATTAATCAACGGTGAACGTGTTGATGCGCTGGCGTTGATCACCCACCGTGATAATTCGCAAAACCGCGGTCGCGAGTTGGTGGAGAAGATGAAAGATCTGATCCCACGCCAGCAGTTTGATATCGCCATTCAGGCAGCGATTGGTACGCACATCATTGCGCGATCCACCGTGAAACAGCTGCGTAAAAACGTACTGGCTAAATGTTATGGCGGCGATATCAGCCGTAAGAAAAAGCTGCTGCAGAAGCAGAAAGAAGGTAAGAAACGCATGAAGCAGATCGGTAACGTCGAGCTGCCGCAGGAAGCGTTCCTCGCCATTCTGCACGTCGGCAAAGACAACAAATAAGGATCCGGCTGCTAACAAAGCCCGAAAGGAAGCTGAGTTGGCTGCTGCCACCGCTGAGCAATAACTAGCATAACCCCTTGGGGCCTCTAAACGGGTCTTGAGGGGTTTTTTG

>EngA fused with His-tagged SUMO protein in pET-15b

TAATACGACTCACTATAGGGGAATTGTGAGCGGATAACAATTCCCCTCTAGAAATAATTTTGTTTAACTTTAAGAAGGAGATATACCATGGGCAGCAGCCATCATCATCATCATCACAGCAGCGGCATGTCGGACTCAGAAGTCAATCAAGAAGCTAAGCCAGAGGTCAAGCCAGAAGTCAAGCCTGAGACTCACATCAATTTAAAGGTGTCCGATGGATCTTCAGAGATCTTCTTCAAGATCAAAAAGACCACTCCTTTAAGAAGGCTGATGGAAGCGTTCGCTAAAAGACAGGGTAAGGAAATGGACTCCTTAAGATTCTTGTACGACGGTATTAGAATTCAAGCTGATCAGACCCCTGAAGATTTGGACATGGAGGATAACGATATTATTGAGGCTCACAGAGAACAGATTGGTGGTATGGTACCTGTGGTCGCGCTTGTCGGGCGCCCTAACGTAGGAAAATCCACGTTATTTAACCGTCTAACTCGCACCCGAGATGCGCTGGTTGCGGATTTCCCGGGTCTGACTCGTGACCGTAAGTACGGTCGTGCGGAAATTGAAGGCCGTGAGTTTATCTGTATTGATACCGGCGGGATTGATGGCACAGAAGACGGTGTAGAAACCCGCATGGCGGAACAGTCGCTGCTGGCGATTGAAGAAGCGGACGTCGTACTGTTTATGGTGGATGCGCGCGCGGGCCTGATGCCGGCAGATGAAGCGATTGCCAAACATCTGCGCTCCCGTGAAAAACCGACCTTCCTGGTGGCAAACAAAACTGACGGTCTGGATCCCGATCAGGCAGTGGTTGATTTCTACTCGCTTGGTTTAGGTGAAATCTACCCGATCGCCGCGTCTCACGGTCGTGGCGTATTAAGTCTGCTGGAGCATGTGCTGCTGCCGTGGATGGAAGATCTCGCACCGCAAGAGGAAGTCGACGAAGACGCTGAATACTGGGCGCAATTTGAAGCGGAAGAGAACGGCGAAGAAGAAGAGGAAGACGACTTCGACCCGCAAAGTCTGCCGATCAAACTGGCGATTGTGGGTCGTCCGAACGTAGGTAAGTCTACACTCACTAACCGTATTCTTGGTGAAGAGCGCGTTGTTGTTTACGACATGCCTGGCACGACGCGTGACAGCATCTACATCCCAATGGAACGCGATGGACGTGAGTATGTGCTCATTGACACCGCTGGCGTACGTAAACGCGGCAAAATCACCGATGCTGTAGAGAAATTCTCCGTAATCAAAACGTTGCAGGCCATTGAAGACGCCAACGTGGTGATGTTAGTGATTGATGCGCGCGAAGGTATTTCCGATCAGGATCTCTCGCTGCTGGGCTTTATTCTCAATAGTGGGCGCTCACTTGTCATTGTGGTGAATAAGTGGGATGGCCTGAGTCAGGAAGTGAAAGAGCAGGTGAAAGAAACGCTGGACTTCCGTCTGGGCTTTATCGATTTTGCTCGTGTGCACTTTATCTCTGCCTTGCACGGCAGTGGTGTTGGTAACTTGTTTGAATCAGTACGTGAAGCGTATGACAGCTCCACCCGTCGTGTGGGGACCTCTATGCTGACGCGCATCATGACGATGGCTGTTGAAGATCACCAACCGCCGCTGGTACGCGGTCGTCGTGTGAAGCTGAAATATGCCCACGCCGGTGGTTATAACCCGCCGATTGTGGTGATTCACGGTAATCAGGTGAAAGACCTGCCTGATTCCTACAAGCGCTACTTGATGAACTACTTCCGCAAATCGCTGGACGTAATGGGATCGCCGATTCGTATTCAGTTCAAAGAAGGGGAAAACCCGTATGCGAATAAGCGTAACACCCTGACGCCAACCCAGATGCGTAAACGTAAGCGTCTGATGAAGCACATCAAGAAAAATAAATAACATATGCCCGGGCTCGAGGGACCCCGCGGGCGGCCGCGTCGACGGATCCGGCTGCTAACAAAGCCCGAAAGGAAGCTGAGTTGGCTGCTGCCACCGCTGAGCAATAACTAGCATAACCCCTTGGGGCCTCTAAACGGGTCTTGAGGGGTTTTTTG

>ObgE fused with His-tagged SUMO protein in pET-15b

TAATACGACTCACTATAGGGGAATTGTGAGCGGATAACAATTCCCCTCTAGAAATAATTTTGTTTAACTTTAAGAAGGAGATATACCATGGGCAGCAGCCATCATCATCATCATCACAGCAGCGGCATGTCGGACTCAGAAGTCAATCAAGAAGCTAAGCCAGAGGTCAAGCCAGAAGTCAAGCCTGAGACTCACATCAATTTAAAGGTGTCCGATGGATCTTCAGAGATCTTCTTCAAGATCAAAAAGACCACTCCTTTAAGAAGGCTGATGGAAGCGTTCGCTAAAAGACAGGGTAAGGAAATGGACTCCTTAAGATTCTTGTACGACGGTATTAGAATTCAAGCTGATCAGACCCCTGAAGATTTGGACATGGAGGATAACGATATTATTGAGGCTCACAGAGAACAGATTGGTGGTATGAAGTTTGTTGATGAAGCATCGATTCTGGTCGTTGCAGGTGATGGCGGTAATGGTTGCGTGAGCTTCCGCCGCGAAAAGTATATTCCGAAAGGCGGCCCGGATGGCGGCGACGGCGGTGATGGTGGTGACGTATGGATGGAAGCCGACGAGAACCTGAACACGCTTATCGATTATCGTTTTGAAAAATCTTTCCGTGCAGAGCGCGGTCAGAATGGCGCAAGCCGCGACTGTACCGGTAAGCGCGGTAAAGACGTGACGATTAAAGTGCCGGTAGGTACGCGTGTAATCGACCAGGGTACTGGTGAAACCATGGGCGATATGACCAAACACGGTCAGCGTCTGCTGGTTGCTAAGGGCGGCTGGCACGGTCTGGGCAATACCCGTTTCAAATCGTCCGTTAACCGTACACCGCGGCAGAAAACCAACGGCACGCCGGGCGATAAGCGCGAGCTGCTGCTGGAGCTGATGCTGCTGGCTGACGTCGGTATGTTGGGGATGCCAAACGCGGGTAAATCGACCTTTATTCGTGCGGTATCGGCGGCTAAACCGAAAGTGGCGGATTATCCGTTTACCACTCTGGTGCCAAGTCTGGGTGTGGTACGAATGGACAACGAAAAGAGCTTCGTTGTTGCCGATATTCCAGGACTGATTGAAGGCGCTGCGGAAGGCGCAGGTCTGGGCATTCGCTTCCTGAAGCACCTGGAACGTTGCCGCGTCCTGTTGCACCTCATCGATATCGATCCGATTGACGGCACCGATCCGGTTGAAAACGCGCGTATTATTATCAGCGAGCTGGAAAAATACAGCCAGGATCTGGCGACGAAACCGCGTTGGTTAGTGTTCAACAAGATCGATCTGCTGGATAAGGTAGAAGCCGAAGAGAAAGCGAAAGCGATCGCTGAGGCGCTGGGCTGGGAAGATAAATATTATCTGATCTCTGCGGCGAGTGGACTGGGCGTGAAAGATCTCTGCTGGGATGTGATGACCTTTATCATTGAAAACCCGGTCGTGCAGGCTGAAGAAGCGAAACAGCCAGAGAAAGTCGAATTCATGTGGGATGATTATCATCGCCAGCAGCTTGAAGAGATTGCTGAAGAGGATGATGAAGACTGGGATGACGACTGGGACGAAGACGACGAAGAAGGCGTTGAGTTCATTTACAAGCGTTAACATATGCCCGGGCTCGAGGGACCCCGCGGGCGGCCGCGTCGACGGATCCGGCTGCTAACAAAGCCCGAAAGGAAGCTGAGTTGGCTGCTGCCACCGCTGAGCAATAACTAGCATAACCCCTTGGGGCCTCTAAACGGGTCTTGAGGGGTTTTTTG

>sfGFP fused with Strep-tag II in the previously constructed vector based on pUC18 (ref. 50 of the manuscript)

TAATACGACTCACTATAGGGAGACCACAACGGTTTCCCTCTAGAAATAATTTTGTTTAACTTTAAGAAGGAGATATACCATGGCTAGCACTTGGTCTCACCCGCAGTTTGAGAAGCTGGAAGTTCTGTTCCAGGGGCCCcatATGAGTAAAGGAGAAGAACTTTTCACTGGAGTTGTCCCAATTCTTGTTGAATTAGATGGTGATGTTAATGGGCACAAATTTTCTGTCCGTGGAGAGGGTGAAGGTGATGCAACAAACGGAAAACTTACCCTTAAATTTATTTGCACTACTGGAAAACTACCTGTTCCATGGCCAACACTTGTCACTACTTTAACTTATGGTGTTCAATGCTTTTCCCGTTATCCGGATCACATGAAACGGCATGACTTTTTCAAGAGTGCCATGCCCGAAGGTTATGTACAGGAACGCACTATATCTTTCAAAGATGACGGGACCTACAAGACGCGTGCTGAAGTCAAGTTTGAAGGTGATACCCTTGTTAATCGTATCGAGTTAAAAGGTATTGATTTTAAAGAAGATGGAAACATTCTCGGACACAAACTCGAGTACAACTTTAACTCACACAATGTATACATCACGGCAGACAAACAAAAGAATGGAATCAAAGCTAACTTCAAAATTCGCCACAACGTTGAAGATGGATCCGTTCAACTAGCAGACCATTATCAACAAAATACTCCAATTGGCGATGGCCCTGTCCTTTTACCAGACAACCATTACCTGTCGACACAATCTGTCCTTTCGAAAGATCCCAACGAAAAGCGTGACCACATGGTCCTTCTTGAGTTTGTAACTGCTGCTGGGATTACACATGGCATGGATGAGCTCTACAAATAAGGATCCCGGGAATTCTCGAGAAGCTTTAGCATAACCCCTTGGGGCCTCTAAACGGGTCTTGAGGGGTTTTTTG

>DHFR fused with Strep-tag II in the previously constructed vector based on pUC18 (ref. 50 of the manuscript)

TAATACGACTCACTATAGGGAGACCACAACGGTTTCCCTCTAGAAATAATTTTGTTTAACTTTAAGAAGGAGATATACCATGGCTAGCACTTGGTCTCACCCGCAGTTTGAGAAGCTGGAAGTTCTGTTCCAGGGGCCCCATATGATCAGTCTGATTGCGGCGTTAGCGGTAGATCGCGTTATCGGCATGGAAAACGCCATGCCGTGGAACCTGCCTGCCGATCTCGCCTGGTTTAAACGCAACACCTTAAATAAACCCGTGATTATGGGCCGCCATACCTGGGAATCAATCGGTCGTCCGTTGCCAGGACGCAAAAATATTATCCTCAGCAGTCAACCGGGTACGGACGATCGCGTAACGTGGGTGAAGTCGGTGGATGAAGCCATCGCGGCGTGTGGTGACGTACCAGAAATCATGGTGATTGGCGGCGGTCGCGTTTATGAACAGTTCTTGCCAAAAGCGCAAAAACTGTATCTGACGCATATCGACGCAGAAGTGGAAGGCGACACCCATTTCCCGGATTACGAGCCGGATGACTGGGAATCGGTATTCAGCGAATTCCACGATGCTGATGCGCAGAACTCTCACAGCTATTGCTTTGAGATTCTGGAGCGGCGGTAAGGATCCCGGGAATTCTCGAGAAGCTTTAGCATAACCCCTTGGGGCCTCTAAACGGGTCTTGAGGGGTTTTTTG
