# Supplementary material for: Single-step in vitro reconstitution of the Escherichia coli ribosome mediated by two GTPase factors, EngA and ObgE
Source: eLife. 2026 Jun 18;15:RP109916. doi: 10.7554/eLife.109916 (PMC13278734; doi:10.7554/eLife.109916)
Supplement: Peer review history

# Editor report: eLife Assessment

**DOI:** 10.7554/eLife.109916.4.sa0

**Authors:** Alan G Hinnebusch

**Affiliations:** Eunice Kennedy Shriver National Institute of Child Health and Human Development United States

**Keywords:** Convincing, Valuable

This is a **valuable** study presenting **convincing** data indicating that the bacterial GTPases EngA and ObgE enable single-step reconstitution of functional 50S ribosomal subunits under near-physiological conditions. The study elegantly bridges the gap between the non-physiological aspects of the previous two-step reconstitution method and the extract-dependent iSAT system to enable assembly of highly functional ribosomes under translation-compatible conditions. The reported findings represent substantial progress towards achieving a bottom-up reconstruction of the translation machinery from synthetic parts.

# Referee report: Reviewer #1 (Public review)

**DOI:** 10.7554/eLife.109916.4.sa1

[Editors' note: this version has been assessed by the Reviewing Editor without further input from the original reviewers. The authors have provided new data and text that addresses all of the reviewers' comments on the previous versions in a wholly satisfactory way.]

Summary:

This study presents evidence that addition of the two GTPases EngA and ObgE to reactions comprised of rRNAs and total ribosomal proteins purified from native bacterial ribosomes can bypass the requirements for non-physiological temperature shifts and Mg+2 ion concentrations for in vitro reconstitution of functional *E. coli* ribosomes.

Strengths:

This advance allows ribosome reconstitution in a fully reconstituted protein synthesis system containing individually purified recombinant translation factors, with the reconstituted ribosomes substituting for native purified ribosomes to support protein synthesis. This represents a significant development in the long-term effort to produce synthetic cells.

# Referee report: Reviewer #2 (Public review)

**DOI:** 10.7554/eLife.109916.4.sa2

This study has developed a single-step method to assemble active bacterial ribosomes under near-physiological conditions by using the GTPase factors EngA and ObgE. These factors eliminate the need for the traditional, harsh manipulations of temperature and magnesium levels. This integration is an important step toward the bottom-up construction of synthetic cells.

# Author comment: Author response

**DOI:** 10.7554/eLife.109916.4.sa3

**Authors:** Aya Sato, Weng Yu Lai, Yusuke Sakai, Keiko Masuda, Yoshihiro Shimizu

**Affiliations:** RIKEN Center for Biosystems Dynamics Research Kobe Japan; RIKEN Center for Biosystems Dynamics Research Kobe Japan; RIKEN Center for Biosystems Dynamics Research Kobe Japan; RIKEN Center for Biosystems Dynamics Research Kobe Japan; RIKEN Center for Biosystems Dynamics Research Kobe Japan

The following is the authors’ response to the previous reviews

**Public Reviews:**

**Reviewer #1 (Public review):**

Summary:

This study presents evidence that addition of the two GTPases EngA and ObgE to reactions comprised of rRNAs and total ribosomal proteins purified from native bacterial ribosomes can bypass the requirements for non-physiological temperature shifts and Mg+2 ion concentrations for in vitro reconstitution of functional *E. coli* ribosomes.

Strengths:

This advance allows ribosome reconstitution in a fully reconstituted protein synthesis system containing individually purified recombinant translation factors, with the reconstituted ribosomes substituting for native purified ribosomes to support protein synthesis. This represents a significant development in the long-term effort to produce synthetic cells.

Weaknesses:

The authors carried out additional experiments indicating that ~60% of the reconstituted ribosomes are functional and that a significant proportion are capable of synthesizing GFP from the correct initiation codon to the correct stop codon, and also of producing an enzymatically active protein at appreciable levels. Their SDS-PAGE and MS analyses of N-terminally tagged GFP are also quite useful but did not assess the frequency of initiation at the wrong start codon, termination at the incorrect stop codon, or the frequency of frameshifting during elongation. This would require examining additional reporters designed to examine dependence on a Shine-Dalgarno sequence or the impact of an in-frame stop codon to assess the fidelity of initiation and termination events, respectively, and one with a programmed frameshift site to assess the elongation fidelity of their reconstituted ribosomes.

In response to the reviewer’s comment, we expanded the MS analysis and performed additional analyses against amino acid sequences corresponding to all three reading frames (updated Supplementary Data 2). As a result, only a single peptide fragment likely derived from the +1 frame was detected, but its intensity was approximately 1/1000 of that of peptide fragments detected from the normal frame. No other out-of-frame peptides were detected, and no evidence of stop-codon readthrough was found. We consider that these results suggest that the kind of deterioration in ribosome function is not occurring in the reconstituted ribosomes. Because this analysis cannot completely rule out abnormal translation events such as initiation from internal start codons or termination at internal stop codons, we also added a statement acknowledging that further analyses will be required to examine all aspects of the translation reaction.

Reconstitution studies in the past have succeeded by using all recombinant, individually purified RPs that, if successful here, would have eliminated the possibility that one or more unknown ribosome assembly factors that co-purify with native ribosomes was added to their reconstitution reactions.

The issue raised by the reviewer was already added at the end of the Discussion in the previous revision. We fully agree with the reviewer’s point and we are currently continuing research in our laboratory aimed at achieving a more fundamental understanding of ribosome assembly.

**Reviewer #2 (Public review):**

This study has developed a single-step method to assemble active bacterial ribosomes under near-physiological conditions by using the GTPase factors EngA and ObgE. These factors eliminate the need for the traditional, harsh manipulations of temperature and magnesium levels. This integration is an important step toward the bottom-up construction of synthetic cells.

Comments on revisions:

The authors have addressed my concerns in the previous round of review.

**Recommendations for the authors:**

**Reviewer #1 (Recommendations for the authors):**

The authors are urged to acknowledge that more sophisticated reporter assays would be required to compare the frequencies of errors occurring at each step of translation using their reconstituted versus native ribosomes.

As described in our response to Reviewer #1, we performed additional MS analyses, updated Supplementary Data 2, and added a statement acknowledging the reviewer’s comment.
